# Artificial Intelligence, Wearable Technologies, and Virtual Reality in Precision Nutrition and Obesity Management: A Critical Narrative Review

**DOI:** 10.3390/diseases14080295

**Published:** 2026-08-14

**Authors:** Yin Yin Bashir, Rahaf AL-Huneiti, Anfal AL-Dalaeen, Firas S. Azzeh

**Affiliations:** 1 Department of Clinical Nutrition and Dietetics, Faculty of Allied Medical Sciences, Applied Science Private University, Amman 11937, Jordan; 2 Preparatory Year Program, Department of Biology, Batterjee Medical College, Jeddah 21442, Saudi Arabia

**Keywords:** artificial intelligence, wearable technologies, virtual reality, obesity, digital health, weight management

## Abstract

Background: Obesity is a chronic, multifactorial disease that demands personalized and sustainable management approaches. Digital health technologies, such as artificial intelligence, wearable devices, mobile health apps, and virtual reality (VR), may support obesity care by providing enhanced behavioral monitoring, personalized feedback, and patient engagement. Objective: This critical narrative review discusses the current evidence on artificial intelligence, wearable technologies, and VR in the context of precision nutrition and obesity management and their possible clinical applications and limitations. Method: A critical narrative review was conducted using peer-reviewed literature published between 2019 and 2026 and identified through PubMed and Google Scholar. Search terms included combinations of “precision nutrition,” “personalized nutrition,” “obesity,” “weight management,” “metabolic health,” “digital health,” “artificial intelligence,” “machine learning,” “mobile health,” “wearable devices,” and “omics” using Boolean operators. Evidence from randomized controlled trials, systematic reviews, meta-analyses, and key conceptual studies was critically synthesized due to substantial heterogeneity in interventions and outcomes. Result: Wearables and mobile applications can enable continuous self-monitoring of physical activity, dietary intake, sleep, and physiological measures. Artificial intelligence may improve dietary personalization, risk prediction, glycemic control, and adaptive feedback. VR offers an immersive way to tackle behavioral and cognitive mechanisms related to overeating such as cravings, food cue reactivity, and inhibitory control. However, the evidence is heterogeneous, with many studies limited by short follow-up periods, small samples, variable adherence, and insufficient clinical validation. Conclusions: Artificial intelligence, wearable technologies, and VR are promising tools for precision obesity management, but their long-term clinical effectiveness remains uncertain. Future research should prioritize adequately powered trials, longer follow-up, standardized outcomes, transparent algorithms, ethical data governance, and integration with multidisciplinary nutrition and obesity care.

## 1. Introduction

Obesity has emerged as one of the most pressing global public health challenges of the twenty-first century, affecting more than one billion individuals worldwide and contributing substantially to premature mortality, disability, and escalating healthcare expenditures [1]. Characterized by excessive adipose tissue accumulation resulting from a complex interaction of genetic, metabolic, behavioral, environmental, psychological, and socioeconomic factors, obesity is now recognized as a chronic, relapsing, multifactorial disease rather than merely a consequence of excessive caloric intake or insufficient physical activity [2]. It is strongly associated with numerous comorbidities, including type 2 diabetes mellitus, cardiovascular disease [3], hypertension, dyslipidemia, non-alcoholic fatty liver disease [4], obstructive sleep apnea, musculoskeletal disorders, reproductive dysfunction, and several obesity-related cancers [5,6,7,8]. Moreover, obesity has profound psychological consequences, including depression, anxiety, impaired body image, reduced self-esteem, social stigma, and diminished quality of life, highlighting the need for comprehensive and individualized management strategies that address both physical and mental health outcomes [9,10].

Lifestyle modification incorporating dietary intervention, increased physical activity, and behavioral therapy remain the cornerstone of obesity prevention and treatment [11]. Clinical guidelines consistently recommend comprehensive lifestyle interventions as first-line therapy because modest and sustained weight loss can significantly improve metabolic health and reduce obesity-related complications [12]. However, despite the proven efficacy of structured lifestyle programs in controlled clinical settings, maintaining long-term adherence remains a major challenge [13]. Many individuals experience declining motivation, poor self-monitoring, limited access to specialized healthcare professionals, environmental barriers, and psychological stressors that ultimately contribute to weight regain after initial success [14,15]. Consequently, long-term weight maintenance remains one of the greatest challenges in obesity management, emphasizing the need for innovative, scalable, and personalized interventions capable of providing continuous support outside traditional healthcare environments.

Recent advances in digital health technologies have transformed the landscape of chronic disease management by enabling continuous monitoring, personalized feedback, remote coaching, and real-time behavioral support [16,17]. Mobile health (mHealth) applications [18], wearable devices [5], telehealth platforms [19], artificial intelligence (AI) [20], and immersive VR technologies are increasingly being integrated into obesity management programs to enhance patient engagement, facilitate self-monitoring, and improve adherence to lifestyle recommendations [21]. These technologies support a shift from episodic clinic-based care toward continuous, patient-centered management that can adapt interventions according to individual behavioral patterns, physiological responses, and environmental contexts.

AI further enhances precision nutrition and obesity management by analyzing large and complex datasets, including lifestyle behaviors, dietary patterns, physiological measurements, and clinical characteristics [16,22]. Machine learning approaches can identify individual risk profiles, predict treatment responses, and generate personalized recommendations to improve adherence and long-term outcomes [20]. Similarly, VR-assisted interventions provide immersive and controlled environments that allow for the assessment and modification of food-related behaviors, physical activity motivation, body image perception, and emotional responses to eating cues [23]. Emerging evidence suggests that VR may serve as a valuable adjunct to conventional behavioral interventions by increasing engagement and facilitating targeted psychological and behavioral strategies [21,24].

Despite these promising advances, several challenges remain, including data privacy concerns, limited long-term clinical evidence, variability in technological platforms, accessibility barriers, and the need for standardized evaluation protocols. Therefore, this narrative review aimed to critically examine the current applications of AI, wearable technologies, and VR in precision nutrition and obesity management. This review summarizes existing evidence, discusses limitations, and identifies future research directions for integrating digital innovations into personalized obesity care.

## 2. Review Approach

This critical narrative review summarizes current evidence on AI, wearable technologies, mobile health (mHealth) applications, and virtual reality (VR) in obesity management and follows general principles for high-quality narrative reviews. Relevant literature published between 2019 and 2026 was identified through searches of PubMed and Google Scholar using combinations of the keywords “precision nutrition,” “personalized nutrition,” “obesity,” “weight management,” “artificial intelligence,” “wearable technologies,” “mobile health,” “virtual reality,” and “digital health” with Boolean operators (AND, OR). Reference lists of selected articles were also screened. The review focused on critical interpretation rather than exhaustive literature retrieval. High-quality evidence, including systematic reviews, meta-analyses, randomized controlled trials, and clinically relevant observational, pilot, and feasibility studies, was prioritized.

Studies were considered relevant if they investigated digital or technology-assisted interventions for obesity, weight management, dietary habits, physical activity, glycemic control, behavior tracking, cognitive retraining, or personalized feedback. Explicit exclusion criteria included non-English publications, animal or in vitro studies, non-peer-reviewed conference abstracts without full-text publications, editorials, letters, commentaries, and articles not directly relevant to the review objectives. Following screening for relevance and quality, 14 peer-reviewed studies were included in the narrative synthesis. The available evidence was heterogeneous in study design, intervention characteristics, populations, intervention duration, and outcome measures; therefore, a formal systematic review methodology or meta-analysis was not undertaken. The evidence was narratively and critically synthesized with a focus on study design, intervention type, population characteristics, clinical outcomes, behavioral mechanisms, technological features, methodological limitations, and translational relevance to clinical nutrition and obesity care. The aim was to provide a comprehensive and balanced overview of the existing evidence, identify knowledge gaps, and discuss future directions for precision obesity management.

## 3. Digital Health and Wearable Technologies in Obesity Management

The increasing prevalence of overweight individuals and obesity has led to the development of digital health technologies to support monitoring, management, and long-term behavior change [16]. Mobile health (mHealth) applications and wearable sensors are increasingly being used to collect behavioral and physiological data, including physical activity, sleep patterns, dietary intake, body weight, heart rate, and user engagement [18,25]. Digital approaches for dietary intake assessment have evolved beyond traditional self-reported food records by incorporating image-based food recognition, automated portion size estimation, barcode scanning, and AI-assisted dietary analysis. Smartphone applications equipped with computer vision algorithms can analyze meal images to identify foods and estimate portion sizes, reducing the burden of manual food logging. In addition, food-tracking applications allow users to record dietary intake, receive personalized feedback, and monitor adherence to dietary goals. Emerging technologies, including smart utensils, connected eating devices, and wearable sensors, can capture eating behaviors such as eating speed, meal timing, and frequency, providing objective measures that may complement conventional dietary assessment methods.

These tools may support patient with self-monitoring, improving communication with their healthcare providers, setting goals, and receiving timely feedback that can encourage lifestyle modifications [18,26]. Clinical and real-world data suggest that digital health tools may contribute to modest improvements in weight-related outcomes, particularly when coupled with behavioral support and feedback. The combination of a smartphone application and a smart band has resulted in greater short-term improvements in body composition and physical activity than standard lifestyle counseling alone among adults who are overweight or obese in the EVIDENT 3 randomized controlled trial [27]. Similarly, app-based multimodal lifestyle interventions have shown clinically meaningful reductions in body weight and improvements in self-monitoring, physical activity, and quality-of-life-related outcomes over periods ranging from 12 weeks to 12 months [28,29]. Wearable-supported interventions may have potential in certain populations, but the evidence remains less robust. Technology-based weight-loss systems and wearable-augmented interventions have shown improvements in physical activity, anthropometric measures, and functional outcomes in adults living with obesity and older adults in rural areas [26,30]. However, these studies are often limited by small sample sizes, short durations of follow-up, and variable adherence, which limit the generalizability of the findings.

**Figure 1:**
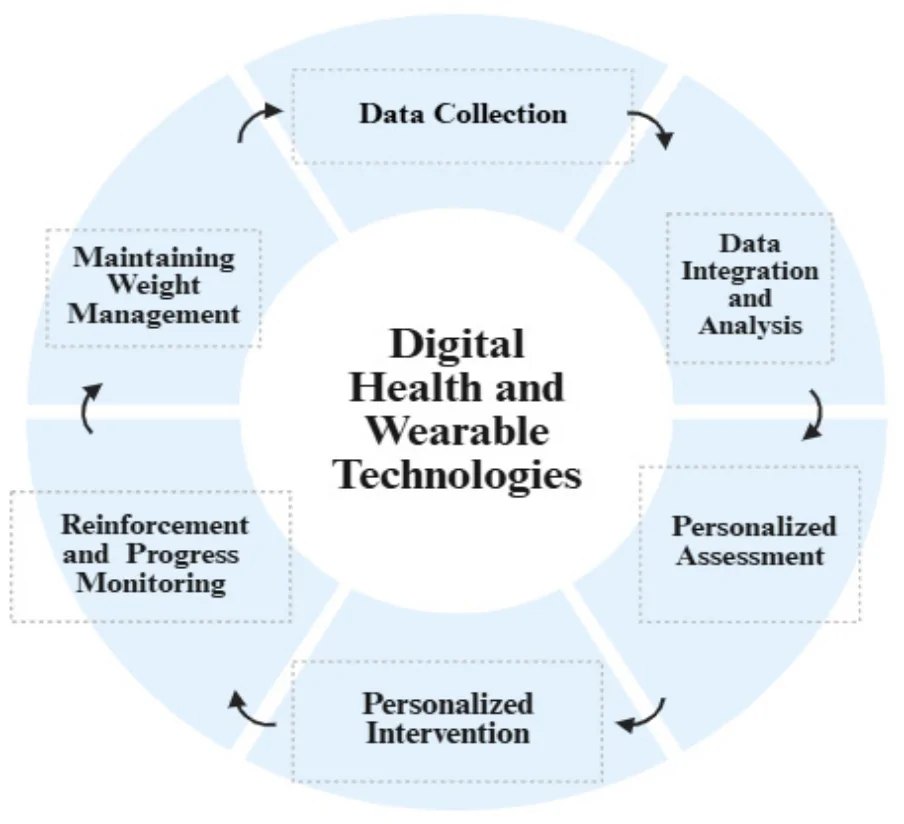
The digital health continuous feedback loop for weight management.

Overall, mobile applications and wearable devices appear most useful when integrated into structured behavioral programs and not as stand-alone tools. Their main contribution is the establishment of a continuous feedback loop in which behavioral and physiological data are collected, interpreted, and converted into personalized goals, professional guidance, and adaptive feedback. As illustrated in Figure 1, this feedback loop can facilitate self-regulation by increasing individuals’ awareness of their daily behaviors, monitoring their progress and allowing more personalized intervention strategies. However, major challenges regarding continuous engagement, data accuracy, privacy protection, and clinical integration must be addressed for these technologies to be implemented in routine obesity care.

This figure illustrates the continuous feedback loop underlying digital weight management interventions. Data are collected through mobile applications and wearable devices, including information on daily steps, sleep duration, and dietary intake. These data are analyzed in relation to personalized goals, enabling professional assessment and the development of tailored intervention strategies. Continuous monitoring and feedback may support self-regulation, goal attainment, and long-term adherence.

## 4. Virtual Reality (VR) as a Tool for Behavioral Change

VR is an immersive digital technology that can create controlled, interactive, and realistic environments for assessment and behavioral intervention. VR may be useful in obesity care because eating behavior is influenced not only by energy balance but also by cognitive, emotional, and environmental responses to food-related cues [17]. Unlike traditional digital interventions that focus on monitoring behaviors such as physical activity, sleep, or dietary intake, VR can expose individuals to simulated food environments and measure responses such as cravings, attentional bias, reaction time, body perception, and inhibitory control [31,32].

**Figure 2:**
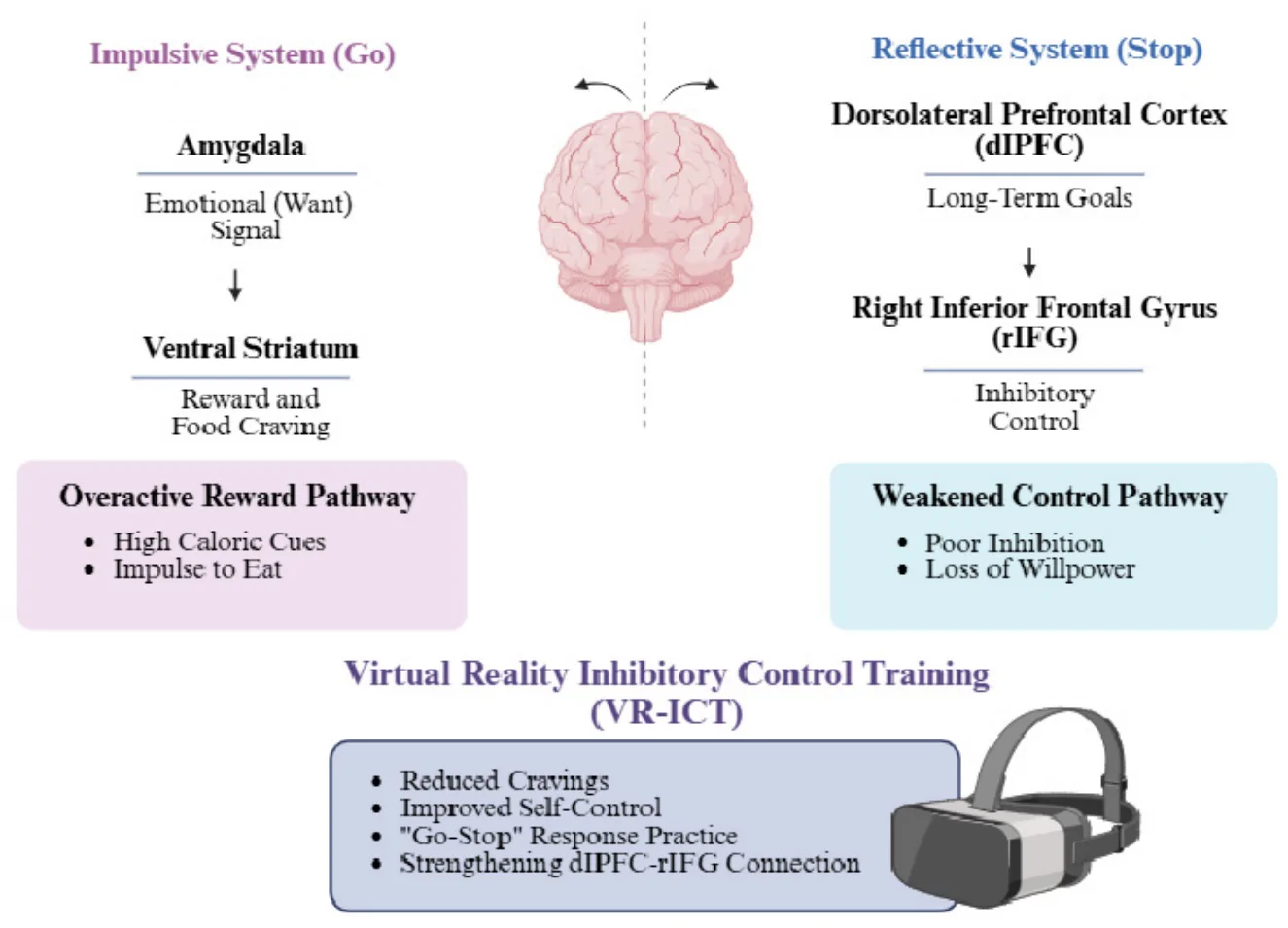
Virtual reality and the brain: impulsive and reflective pathways in eating control. This figure illustrates the impulsive and reflective pathways involved in eating behavior and the potential role of virtual reality (VR) in targeting these mechanisms. The impulsive pathway is associated with reward-related and emotional responses to food cues, involving brain regions such as the amygdala and ventral striatum, which may contribute to cravings and automatic approach behaviors toward high-calorie foods. In contrast, the reflective pathway involves regions associated with executive function and inhibitory control, including the dorsolateral prefrontal cortex (dlPFC) and right inferior frontal gyrus (rIFG), which support goal-directed behavior and self-regulation. In individuals who are overweight or obese, dysfunction in the balance between these pathways may contribute to overeating and impaired inhibitory control. VR-based interventions may provide controlled environments for food cue exposure and inhibitory control training, potentially supporting improved self-regulation and eating behavior.

Dual-system models of eating behavior provide a partial framework for understanding the possible role of VR in obesity management. These models involve an interaction between a fast, impulsive system that automatically reacts to rewarding food cues, and a slower, reflective system that is involved in self-regulation and inhibitory control [33,34]. In individuals who are overweight or obese, exposure to highly palatable food cues may enhance reward-related responses and diminish the capacity to inhibit automatic approach behaviors [35,36]. This interpretation is consistent with meta-analytic evidence on executive function deficits among individuals who are overweight and obese, particularly regarding deficits in inhibition and working memory [37]. This imbalance may contribute to overeating, cravings, and difficulty maintaining long-term dietary goals. These cognitive and behavioral pathways and the potential role of VR in modulating responses to food-related cues are summarized in Figure 2.

VR may offer a practical platform for targeting these mechanisms safely and in a controlled manner. This is clinically relevant because inhibitory control, commonly assessed using Go/No-Go and Stop-Signal tasks, has been associated with food consumption and food choice in systematic reviews and meta-analytic evidence [38]. VR-based inhibitory control training can use tasks such as Go/No-Go, Stop Signal, or antisaccade paradigms in realistic food settings to train responses to high-calorie food cues. A proof-of-concept study on VR-based inhibitory control training showed feasibility, acceptability, and reductions in loss-of-control eating episodes, but these findings are preliminary, given the small sample size and short intervention period [39]. VR-based cue exposure can also be employed to assess or reduce food-related reactivity by presenting personalized food cues and monitoring emotional and physiological responses [32].

Besides cognitive mechanisms related to eating, VR may also enable physical activity and behavioral engagement. VR-based exercise programs and interactive virtual environments can improve motivation by making physical activity more enjoyable, especially for adolescents or people with barriers to conventional exercise [21]. Moreover, behavioral and psychological interventions related to obesity are being investigated using holistic VR platforms that incorporate avatar embodiment, virtual coaching, and real-time feedback [40].

Overall, VR is a promising method for treating obesity, but the field remains in an early stage. Its main value is in simulating real-life environments, testing behavioral responses under controlled conditions, and targeting cognitive mechanisms such as food-cue reactivity, cravings, body image disturbance, and inhibitory control. However, the existing evidence is still limited by small sample sizes, short follow-up periods, heterogeneity of intervention designs, and a lack of long-term clinical outcomes. There is recent systematic review evidence suggesting potential benefits of VR-enhanced interventions for weight-related and body image outcomes, but further well-designed, randomized controlled trials are required before VR can be considered an established component of routine obesity care [41].

## 5. Artificial Intelligence Applications in Weight Management and Glycemic Control

AI refers to the use of computational methods to perform tasks that are normally attributed to human cognitive processes, such as learning, pattern recognition, prediction, decision support, and adaptive feedback. AI has attracted increasing attention in obesity and metabolic care as it can analyze large and multidimensional datasets generated by mobile applications, wearable sensors, continuous glucose monitoring systems, dietary records, and user-interaction patterns [2]. In contrast to traditional lifestyle interventions that are usually based on periodic clinical visits and general recommendations, AI-supported systems may provide more dynamic and individualized feedback based on user behavior, physiological data, and treatment progress.

AI in the care of people with obesity can be broadly classified into three main areas: personalized nutrition, metabolic monitoring, and educational/communication support. AI-powered digital platforms have been used to facilitate dietary tracking, glucose monitoring, algorithm-based feedback, and personalized recommendations [2,16,42]. For example, digital platforms integrating personalized dietary decision-making with continuous glucose monitoring have been investigated to support metabolic self-management and improve glycemic control in individuals with type 2 diabetes [16]. Real-world studies have also reported improvements in weight-related and glycemic outcomes, and these findings have also been reported by digital health apps that integrate wearable and behavioral data, but causality is limited as many of the studies are observational or short-term [6,42]. AI has also been explored as a tool to support health literacy and clinical communication. AI-programmed robotic systems have facilitated the weight-management decision-making process and health literacy among individuals living with being overweight or obese [7]. Moreover, AI-powered virtual human interventions have been studied as educational tools to improve the obesity-related communication skills of general practitioners s [43,44]. These applications are clinically relevant because effective obesity care involves patient-centered communication, reductions in stigma, and support for shared decision-making, in addition to dietary and behavioral recommendations.

Emerging evidence also suggests that generative AI may hold promise in dietary planning and nutrition education. Studies on AI-generated meal plans, including those generated by ChatGPT (4.0)-based systems, suggest that AI may generate dietary recommendations comparable to human-produced plans in certain contexts [45].

However, because the accuracy, safety, personalization, cultural appropriateness, and suitability of AI-generated dietary advice vary for people with comorbidities, allergies, pregnancy, eating disorders, or complex medical needs, these findings should be interpreted with caution. AI should not replace qualified nutrition professionals but may serve as a supportive tool in supervised clinical nutrition practice. Overall, AI can facilitate the evolution of precision obesity management by converting behavioral and physiological data into predictive insights, personalized feedback, and adaptive intervention strategies. However, evidence remains heterogeneous, and many studies are limited by short durations, variable adherence, limited external validation, and a lack of assessment of long-term weight maintenance. Future studies should focus on transparent algorithms, clinically meaningful outcomes, long-term follow-up, patient safety, data privacy, and integration of AI tools into multidisciplinary obesity and nutrition care. A summary of digital health and artificial intelligence applications in obesity and nutrition management is presented in Table 1.

## 6. Integrated Digital Technologies for Weight Loss and Metabolic Control

The reviewed evidence suggests that digital health technologies may support weight loss and metabolic control through complementary mechanisms. Mobile applications and wearable devices primarily contribute to continuous monitoring, self-reporting, feedback, and behavioral accountability, while AI-based systems may enhance prediction, dietary personalization, glucose monitoring, and adaptive feedback [6,16]. In contrast, VR may offer immersive environments to target behavioral and cognitive mechanisms involved in eating behavior, such as food-cue reactivity, cravings, body image disturbance, and inhibitory control [31,32,39,47]. The integration of these technologies could result in a more adaptive model of obesity care. Wearable devices and mobile applications can produce real-time behavioral and physiological data, AI can analyze these data to discover patterns and personalize recommendations, and VR can be used to deliver targeted behavioral or cognitive interventions. This combination may reduce decision burden, increase engagement, and support more individualized intervention strategies. For example, AI-supported feedback may assist with dietary choice and glycemic self-management, and VR-based exposure or inhibitory control training may be used to target food-related cravings and impulsive eating responses [6,16,32,39].

Mobile health applications further enhance digital weight management by providing platforms for dietary tracking [48], goal setting [49], education [50], behavioral coaching, and communication with healthcare professionals [51]. Advanced applications increasingly incorporate AI-powered nutritional analyses, automated feedback, and personalized meal recommendations based on individual dietary preferences, metabolic profiles, and health objectives [52]. Unlike traditional dietary interventions that often provide static recommendations, digital systems can dynamically modify nutritional advice according to real-time behavioral changes and physiological responses [20]. This adaptive approach may improve dietary adherence and support long-term lifestyle modification.

The combination of wearable technologies and digital coaching has demonstrated promising effects on weight reduction and metabolic outcomes [5]. Studies evaluating technology-assisted lifestyle interventions have reported improvements in body weight, body mass index, physical activity, glycemic control, and cardiometabolic risk factors [6,20,53]. However, evidence suggests that the greatest benefits are achieved when digital tools are combined with human support, such as healthcare professional guidance, behavioral counseling, or structured intervention programs [54]. This highlights the importance of integrating technology as an enhancement rather than a replacement for clinical expertise.

VR provides an additional behavioral component within integrated digital systems by addressing the psychological and cognitive factors that influence obesity. When combined with wearable monitoring and AI-driven personalization, VR can create adaptive environments for practicing healthy eating behaviors, managing food cravings [55], improving body image [56], and increasing motivation for physical activity [11]. For example, physiological responses measured through wearable sensors during VR exposure to food-related environments could be analyzed by AI algorithms to identify emotional eating patterns and personalize behavioral interventions [56].

However, the clinical effectiveness of the integrated model is not clear. Many existing studies have short intervention durations, small or selective samples, heterogeneous technologies, and variable adherence. Some studies examined app-based or wearable-supported interventions over several months, while others were feasibility studies, proof-of-concept studies, or study protocols and did not complete effectiveness trials [21,27,39]. These limitations make it difficult to know whether the improvements observed were due to the technology alone, to the behavioral support provided, to the improved self-monitoring, or to the motivation of the participants.

Another major limitation is long-term maintenance. Digital interventions may initially increase engagement through novelty, frequent feedback, and simplified decision-making, but, over time, sustained use may decline. Limited long-term follow-up data are available and weight regain after digital interventions has been reported in extended follow-up studies [57]. Future research should thus assess short-term weight loss and metabolic outcomes as well as long-term adherence, weight maintenance, patient safety, usability, and integration into standard nutrition and obesity care.

From a clinical nutrition standpoint, AI, wearables, and VR should be considered as adjuncts to professional care and not as replacements for it. AI-generated recommendations may need oversight to ensure they are nutritionally adequate, culturally appropriate, medically safe, and suitable for individuals with comorbidities or complex dietary needs. Likewise, wearable and VR data need to be interpreted in the context of a broader clinical picture that includes dietary assessment, behavioral counseling, metabolic risk, psychological factors, and patient preferences. Future research should therefore focus on rigorous randomized controlled trials, transparent algorithms, standardized outcomes, longer follow-up, and multidisciplinary implementation models.

Future research should focus on optimizing the integration of AI, wearable technologies, VR, and precision nutrition approaches to develop more personalized and sustainable obesity management strategies. Although current digital interventions demonstrate promising effects on weight control, physical activity, and metabolic health, additional large-scale and long-term randomized controlled trials are required to confirm their clinical effectiveness and cost-effectiveness. Future AI-based systems should incorporate diverse data sources that include dietary behaviors, physical activity, sleep patterns, continuous metabolic monitoring, gut microbiome profiles, and psychosocial factors to provide adaptive and individualized recommendations.

Advances in wearable sensors and biosensing technologies may enable more accurate real-time monitoring of metabolic changes and early identification of factors contributing to weight gain or treatment failure. Similarly, VR-based interventions should be further investigated as personalized behavioral therapies targeting food cravings, emotional eating, body image, and motivation for lifestyle change. Future studies should also address challenges related to data privacy, algorithm transparency, digital accessibility, and integration into routine clinical practice. Ultimately, combining digital technologies with multidisciplinary healthcare approaches may facilitate a transition toward proactive, precision-based, and sustainable obesity prevention and management.

## 7. Conclusions

Available evidence suggests that digital health technologies such as AI, wearable devices, mobile health applications, and VR may serve as adjuncts in precision obesity and metabolic management. These technologies may provide continuous monitoring, personalized feedback, behavioral engagement, dietary support, and cognitive or behavioral retraining. However, the clinical effectiveness of these technologies varies, and evidence is limited by heterogeneity in study designs, short follow-up periods, small sample sizes, and limited assessment of long-term weight maintenance. AI may enhance prediction, personalization, and adaptive feedback, and wearable technologies may promote self-monitoring and behavioral accountability. VR, while still an emerging approach, may help target food cue reactivity, cravings, body image disturbance, and inhibitory control, although its use in obesity care remains in an early stage. Future studies should focus on adequately powered randomized controlled trials, standardized outcome measures, longer follow-up periods, algorithmic transparency, and patient safety and their integration into multidisciplinary nutrition and obesity care. These technologies should be considered adjuncts to, rather than substitutes for, professional clinical and nutritional management.

## Figures and Tables

**Table 1:** Summary of digital health, artificial intelligence, wearable, and virtual reality interventions relevant to obesity and metabolic management.

Author Name/Years	Design	Participants	Digital Technology	Result	Duration
Veluvali et al. (2025) [6]	Observational cohort/real-world digital health study	944	January V2 app with CGM and heart-rate monitoring	Reported improvements in weight-related outcomes, time in range, and glycemic control	14 days
Ryan et al. (2025) [44]	Pilot feasibility study	22	AI-driven virtual human	Improved general practitioners’ obesity-related education and communication skills	4 months
Kim et al. (2024) [45]	Qualitative evaluation	95 invited; 67 completed surveys	ChatGPT-generated weight-loss diet plans	Experts had difficulty distinguishing AI-generated from human-created plans; limitations included insufficient specificity and clinical tailoring	11 days
Gemesi et al. (2024) [29]	RCT	168	App-based lifestyle intervention with personalized feedback	Clinically meaningful weight loss at 12 weeks, partly maintained at 24 weeks; improved lifestyle behaviors	24 weeks
Zahedani et al. (2023) [42]	RCT	2217	Season of Me/digital app integrating wearable and behavioral data	Reported weight loss, increased physical activity, and improved glycemic-related outcomes	28 days
Wu et al. (2023) [21]	RCT study protocol	220 planned	VR-based physical exercise training	Planned outcomes include physical activity, BMI, and related health outcomes	8 weeks
Roth et al. (2023) [28]	RCT	150	App-based multimodal weight-loss program (Zanadio)	Significant and clinically relevant body weight reduction; improved self-monitoring and physical activity	12 months
Chu et al. (2022) [17]	Quantitative	144	AI robot developed for this study	Supported weight-management decision-making, health literacy, and shared decision-making	3 months
Anastasiadou et al. (2022) [40]	RCT study protocol	96	VR platform integrating multiple therapeutic components	Designed to evaluate a holistic VR tool for obesity-related behavioral and psychological intervention	12 months
Batsis et al. (2021) [26]	Feasibility study	28	Wearable-augmented weight-loss intervention/Fitbit Flex 2	Feasible intervention; reported improvements in physical activity and functional or anthropometric outcomes	12 weeks
Manasse et al. (2021) [39]	PES	14	VR-ICT system	Feasible and acceptable; reductions in loss-of-control eating episodes	2 weeks
Silberman et al. (2020) [46]	Observational cohort	683	Digital weight-management program with one-on-one coaching	Reported weight loss outcomes in a digital coaching program	12 months
Park et al. (2020) [16]	RCT	294	AI-based dietary management with real-time CGM	Investigated AI-supported dietary management and glucose monitoring for diabetes care	48 weeks
Lugones-Sánchez et al. (2020) [27]	Multicenter RCT	440	Smartphone app and smart band	Improved body composition and physical activity; moderate effect on BMI	3 months

RCT: Randomized Controlled Trial; VR: Virtual Reality; BMI: Body Mass Index; PES: Pilot Experimental Study; VR-ICT: Virtual Reality–Inhibitory Control Training.

## Data Availability

No new data were created or analyzed in this study. Data sharing is not applicable to this article.
